# Comparative efficacy of different growth hormone supplementation protocols in improving clinical outcomes in women with poor ovarian response undergoing assisted reproductive therapy: a network meta-analysis

**DOI:** 10.1038/s41598-024-53780-z

**Published:** 2024-02-09

**Authors:** Zheyun Xu, Weiquan Tong, Ze Yang, Hongyan Zhang, Xingbei Chen

**Affiliations:** 1 Zhejiang Chinese Medical University, Hangzhou, China; 2 Department of Gynecology and Obstetrics, The First Affiliated Hospital of Zhejiang Chinese Medical University (Zhejiang Provincial Hospital of Chinese Medicine), Hangzhou, China; 3 Department of Emergency Medicine, The Third Affiliated Hospital of Zhejiang Chinese Medical University, Hangzhou, China; 4 Department of Reproductive Medicine Center, Hangzhou Women’s Hospital, Hangzhou, China

**Keywords:** Endocrinology, Reproductive disorders

## Abstract

Growth hormone (GH) has a long-standing history of use as an adjunctive therapy in the treatment of poor ovarian response (POR), but the optimal dosage and timing remains unclear. The aim of this study was to evaluate and compare the efficacy of different GH supplementation protocols through a network meta-analysis (NMA) and determine the optimal treatment protocol. This study was reported based on the Preferred Reporting Items for Systematic Reviews for Network Meta-Analysis (PRISMA-NMA) statement. Databases including PubMed, Web of Science, Cochrane Library and Embase were searched until June 2023. A total of 524 records were retrieved in our search, and 23 clinical studies comprising 4889 cycles were involved. Seven different GH protocols were identified. Results showed that compared to the control group, daily administration of 4–8 IU of GH during the follicular phase of the stimulation cycle had the best comprehensive therapeutic effects on improving the number of retrieved oocytes, mature oocytes, endometrial thickness, and reducing gonadotropin requirements in POR patients undergoing assisted reproductive therapy, with a relatively brief treatment duration and a moderate total GH dose. Subgroup analysis demonstrated that this protocol could significantly improve the clinical pregnancy rate of POR patients in the randomized controlled trials (RCT) subgroup and the African subgroup. Therefore, its clinical application is suggested. Besides, the potential advantages of long-term GH supplementation protocol (using GH for at least 2 weeks before oocyte retrieval) has merit for further research. Rigorous and well-designed multi-arm RCTs are needed in the future to confirm the conclusions drawn from this study.

## Introduction

In recent years, more women are effectively conceiving as a result of the rapid development of assisted reproductive technology (ART). However, poor ovarian response (POR), a pathological condition in which the ovary is unable to develop an adequate number of mature follicles in response to normal gonadotropin (Gn) stimulation, remains one of the major challenges of ART therapy^1^. Epidemiologic data show that the prevalence of POR is 9–24% in patients undergoing ovarian stimulation for in vitro fertilization (IVF)^2^. The etiology of POR has not been elucidated yet. It is acknowledged that decline in ovarian reserve caused by aging, genetic factors, ovarian surgery, and certain pelvic infections, is closely related to POR^3^. Pregnancy rate remains low in patients with POR despite a plethora of interventions, which may be attributed to insufficient oocytes at retrieval, insufficient high-quality embryos for transfer, and poor endometrial receptivity^1,4^.

The management and treatment of patients with POR is still a controversial issue in ART. Refining controlled ovarian hyperstimulation (COH) protocols and incorporating adjunctive medications are main approaches currently used in the treatment of POR^5–8^. Nowadays, many drugs are administrated as adjuvant therapy for POR, such as growth hormone (GH), dehydroepiandrosterone (DHEA), coenzyme Q10 (Co Q10), testosterone, estrogen, letrozole, clomiphene, human chorionic gonadotropin (hCG), and recombinant luteinizing hormone (rLH)^7,8^. However, no addition of adjuvants is recommended for PORs before and/or during COH to date, according to the recent ESHRE guideline of ovarian stimulation for ART^9^. Hence, reproductive endocrinologists are still working hard to find evidence of the efficacy of those medications in order to better promote their clinical applications.

GH is a peptide hormone mainly secreted from the pituitary gland^10^. As a key factor in the processes of cell growth, proliferation, and metabolism^11^, its value in reproduction and infertility has drawn much attention since it was first used as an adjuvant to Gn in 1988^12^. During the past several decades, the involvement of GH in reproduction and infertility has been extensively studied. Previous researches indicated that GH may modulate reproductive function by binding to GH receptor directly or by promoting the secretion of insulin-like growth factors (IGFs, the major secondary messengers of GH) to play roles in ovarian steroidogenesis, early follicular recruitment, follicular development, oocyte nucleus maturation, preventing follicular atresia, corpus luteum generation and improving endometrial receptivity^13–16^. Therefore, it is considered biologically plausible that GH supplementation can bring benefits to people undergoing IVF, especially in cases with diminished ovarian reserve, who are at risk of experiencing POR^14^.

Although GH has been used in female infertility for over thirty years, there is still a lack of guidelines or consensus on the administration regimen of GH, so clinical protocols are always based on the experience of ART centers or individual clinicians, resulting in significant distinctions in the timing and dosage of administration. Considering the substantially higher cost of GH administration and its potential health risk^17^, it appears necessary to determine an appropriate dosage and administration schedule to prevent overdose and reduce patients' medical expenses. Besides, the effects of GH on clinical pregnancy rate and live birth rate in patients with POR remains unclear^18–20^, and whether it is related to the discrepancy of GH regimens is uncertain. In addition, previous meta-analyses focusing on this topic demonstrated a high degree of heterogeneity^18,21,22^, possibly attributable to variations in GH supplementation regimens, inconsistent diagnostic criteria (for example, the Bologna criteria^23^, the Poseidon criteria^24^ and diverse self-defined criteria), and other potential confounders, which may affect the strength of the evidence and influence clinical decision-making. All mentioned problems indicate the necessity of exploring differences in efficacy among different GH regimens (including dose and time of administration), in order to provide more evidence for the precise application of GH in patients with POR.

Network meta-analysis (NMA) is a feasible solution to compare the differential effects of multiple interventions and ranking them according to the predicted efficacy by analyzing the results of both direct and indirect comparisons, which can provide important evidence in developing clinical guidelines^25^. Hence, we performed a NMA to compare the effects of different GH supplementation protocols on the clinical outcomes of patients with POR undergoing ART therapy. Besides, subgroup analyses were further conducted to explore the main source of heterogeneity among the studies.

## Materials and methods

### Protocol registration

This NMA was conducted according to the Preferred Reporting Items for Systematic Reviews and Meta-analyses for Network Meta-analyses (PRISMA-NMA)^26^. This review protocol was registered in the PROSPERO database before data extraction (CRD42022170119).

### Literature search strategy

Databases including PubMed, Web of Science, Cochrane Library and Embase were searched by computer from January 1985 to June 2023. The main search terms were as follows: (“growth hormone” or “pituitary growth hormone” or “somatotropin”) and (“in vitro fertilization” or “intracytoplasmic sperm injection” or “test-tube fertilization” or “embryo transfer” or “IVF” or “ICSI”) and (“poor/low ovarian response/responder” or “POR”). Keywords were searched as both subject terms and free terms. References of eligible articles and related meta-analysis were reviewed manually to avoid missing any relevant literature.

### Inclusion and exclusion criteria

Inclusion criteria were as follows: (a) Study design: randomized controlled trials (RCTs) or cohort studies; (b) Language restriction: English; (c) Target population: women with POR undergoing IVF or intracytoplasmic sperm injection (ICSI) treatment; (d) Intervention: the intervention groups must be treated with GH and the control groups be treated without GH or with placebo. (e) Outcomes measures: the primary outcome was the clinical pregnancy rate. The second outcomes were the total dose of Gn for ovarian stimulation, number of retrieved oocytes, number of retrieved mature oocytes (MII oocytes), number of fertilized embryos, serum estradiol (E2) levels on hCG day, endometrial thickness on hCG day, and live birth rate. Clinical pregnancy rate (or live birth rate) was defined as the number of clinical pregnancies (or live births)/the number of embryo transfer cycles * 100%. If the number of embryo transfer cycles was not mentioned, clinical pregnancies (or live birth rate)/number of start cycles*100% was used instead. The continuous data should be reported as mean and standard deviation (SD).

Exclusion criteria were as follows: (a) The details of POR diagnostic criteria or treatment protocol were not reported; (b) Primary outcomes were missing; (c) GH combination with other adjuvant drugs; (d) The data were confusing but the authors could not be contacted; (e) Self-controlled studies, case reports, reviews, comments and replies.

### Literature selection and data extraction

Literature selection and data extraction were conducted by two independent researchers (Z.Y.X. and W.Q.T.). Any disagreement was solved by the third researcher (Z.Y.). The title and abstract were scanned first and after excluding irrelevant references, the full text was read to determine whether eligible. If necessary, we would contact the authors by e-mail to obtain important missing information. Study characteristics involving general information, demography information of patients, intervention, major information of methodological quality assessment, and endpoint outcome measures were extracted from each article.

### Methodological quality assessment

Methodological quality assessment was performed by two independent researchers (Z.Y.X and W.Q.T). RCTs and cohort studies were assessed by the Cochrane Risk of Bias Tool for randomized controlled trials^27^ and the Newcastle–Ottawa Scale (NOS)^28^, respectively. Any disagreement was solved by the third researcher (Z.Y.).

### Statistical analysis

A NMA based on the Bayesians was performed, using multiNMA and geMTC packages in R (Version 4.2.3), to simultaneously compare the curative effect of different GH supplementation protocols for the adjuvant treatment of POR. A random effects model was used for data synthesis. Continuous data were summarized as mean difference (MD) with 95% credible interval (95%Crl), and dichotomous data as odds ratio (OR) with 95%Crl. Consistent and inconsistent models were both employed for NMA to analyze global inconsistency. If the difference in deviation information criterion (DIC) values between the two models was less than 5, the consistent model was chosen; otherwise, the inconsistent model was chosen. We also performed pairwise comparisons if direct data were available and used the node-splitting method to estimate local inconsistency. The efficacy of each intervention was predicted according to the surface under the cumulative ranking curve (SUCRA), with a larger area indicating better efficacy. Publication bias was analyzed graphically by funnel plots using the NMA package in Stata (Version 14.0). Subgroup analyses were completed using the Metan command in Stata.

## Results

### Eligible studies and characteristics

**Figure 1:**
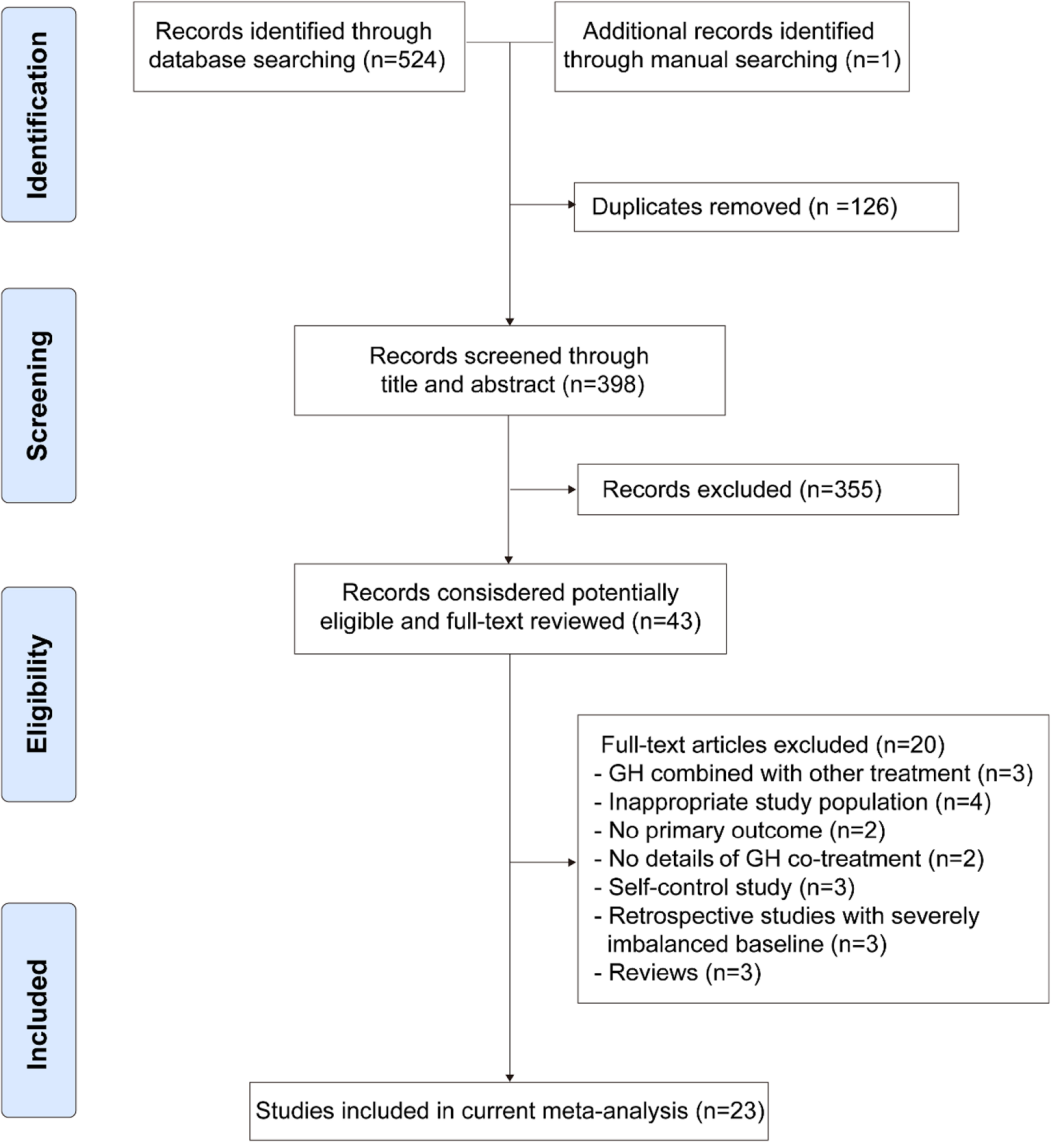
Flow diagram of study selection.

A total of 524 original articles were retrieved. After a series of screening steps, 15 RCTs and 8 cohort studies (comprising a total of 4889 treatment cycles) were finally included^29–51^ (Fig. 1). The characteristics of the eligible studies are presented in Table 1.

**Table 1:** Baseline characteristics of the included studies.

Author and published year	Study type	Number of participants (n) GH + /GH-	Diagnostic criteria	COH protocol	Mean daily dose of GH (IU)	Administration time of GH	Outcomes
Owen^29^ 1991	RCT	13/12	Self-defining criteria	GnRHa short	12(24, qod)	Day1 of stimulation to hCG day	①⑧
Bergh^30^ 1994	RCT	9/29	Self-defining criteria	GnRHa long	About 6 (0.1/kg)	Group A: Follicular phase of stimulation cycle; Group B: 7 days of previous cycle + Follicular phase of stimulation cycle; Group C: 7 days of previous cycle	①③
Dor^31^ 1995	RCT	7/7	Self-defining criteria	GnRHa short	About 6 (18/day, for 4 times)	Day 2, 4, 6, 8 of stimulation cycle	①⑧
Suikkari^32^ 1996	RCT	6/16	Self-defining criteria	GnRHa short	Group A: 4 Group B: 12	Follicular phase of stimulation cycle	①
Kucuk^33^ 2008	RCT	31/30	Self-defining criteria	GnRHa long	12	Day 21 of preceding cycle to hCG day	①②④⑤⑥
Eftekhar^34^ 2012	RCT	40/42	Self-defining criteria	GnRHant	12	Day 21 of preceding cycle to hCG day	①②③④⑤⑥⑦
Hu^35^ 2014	Non-RCT	102/287	The Bologna criteria	GnRHa long	4	Day1 of stimulation to hCG day	①②③④⑤⑥⑦
Bayoumi^36^ 2015	RCT	84/88	The Bologna criteria	Microflare stimulation	7.5	Day 6 of stimulation to hCG day	①②③④⑤⑥⑦
Dunne^37^ 2015	Non-RCT	14/28	The Bologna criteria or self-defining criteria	Micro GnRHa	10	From luteal phase of preceding cycle for 14 successive days	①⑥
Bassiouny^38^ 2016	RCT	68/73	The Bologna criteria	GnRHant	7.5	Day 6 of stimulation to hCG day	①②③④⑤⑥⑦⑧
Ho^39^ 2017	Non-RCT	33/33	Self-defining criteria	GnRHa long	2	Day 3 of stimulation to hCG day	①③④⑥
Choe^40^ 2017	RCT	62/65	The Bologna criteria	GnRHant	About 8.5 (sustained‑release)	Mid-luteal, late luteal of preceding cycle and day2 of stimulation cycle (for 3 times)	①②③④⑥⑦
Chu^41^ 2018	Non-RCT	61/71	The Bologna criteria	Mild stimulation	2.25 (4.5 qod)	From day 16 of preceding cycle for 6 times and from day 1 of stimulation for 3 times	①③⑧
Dakhly^42^ 2018	RCT	120/120	The Bologna criteria	GnRHa long	7.5	Day 21of preceding cycle to hCG day	①②③④⑤⑥⑦⑧
Lee^43^ 2019	Non-RCT	94/90	The Bologna criteria	GnRHa ultralong	About 3.33 (4,4,2)	For 3 successive days, along with ovulation stimulation	①③⑥
Cai^44^ 2019	Non-RCT	338/338	The Poseidon Criteria	GnRHa long or GnRHant	2	Day 2–3 of preceding cycle to ovum pick-up	①②③⑤⑧
Norman^45^ 2019	RCT	62/51	Self-defining criteria	GnRHant	12	Day 1 of stimulation cycle to hCG day	①②③⑧
Safdarian^46^ 2019	RCT	70/35	The Bologna criteria	GnRHant	Group A: 7.5; Group B:0.3	Group A: day 8 of stimulation cycle to hCG day; Group B: day 3 of preceding cycle to hCG day	①②③④⑤⑦⑧
Zhu^47^ 2020	Non-RCT	557/1231	The Poseidon Criteria	Multifarious protocols	4	Day1 of stimulation to hCG day	①③⑦⑧
Gong^48^ 2020	RCT	52/53	The Bologna criteria	GnRHant	4	Day 2 of preceding cycle to hCG day	①②③④⑤⑥⑦
Mohammad^49^ 2021	RCT	78/78	Self-defining criteria	GnRHant ultrashort	4	Day 2 of stimulation cycle to 1 day before ovum pickup	①③④⑤⑥⑦
Zafardoust^50^ 2022	RCT	61/57	The Bologna criteria	GnRHant	15	Day 21 of preceding cycle to hCG day	①②④⑧
Bender^51^ 2022	Non-RCT	47/46	The Bologna criteria	GnRHant	4 (12, every 3 days)	Start in the mid-luteal phase of preceding cycle until the antagonist administration start	①②③⑧

①Clinical pregnancy rate, ②Total dosage of Gn required for ovarian stimulation, ③Number of retrieved oocytes, ④Number of retrieved MII oocytes, ⑤Number of fertilized embryos, ⑥Serum E2 Levels on hCG Day, ⑦Endometrial thickness on hCG Day, ⑧Live birth rate. *GnRHa* Gonadotropin releasing hormone agonist, *GnRHant* Gonadotropin releasing hormone antagonist.

According to the GH supplementation protocols reported in the literature, the daily dose of GH ranged from 0.3–15 IU/d (the average value was 6.78 IU/d, and the first and second tertiles were 4 IU/d and 8.5 IU/d, respectively). The administration time of GH ranged from the early follicular phase of the previous menstrual cycle to the mid-follicular phase of the stimulation cycle. Based on the mean and tertiles of the daily dose of GH, we divided the GH daily dose into three groups: low, medium, and high. Furthermore, considering the administration time of GH, we categorized the GH supplementation protocols into seven types (Protocol A-G). The categories are presented in Table 2.

**Table 2:** Summary of GH supplementation protocols.

GH supplementation protocol	Daily dose of GH(IU/day)	Administration time of GH	Number of studies
Low dose, follicular phase protocol (A)	Less than 4	From follicular phase of the stimulation cycle to hCG day	2
Low dose, long-term protocol (B)		For at least 2 weeks, always from preceding cycle to hCG day	3
Medium dose, luteal phase protocol (C)	4–8	Only luteal phase of preceding cycle	1
Medium dose, follicular phase protocol (D)		From follicular phase of the stimulation cycle to hCG day	9
Medium dose, long-term protocol (E)		For at least 2 weeks, always from preceding cycle to hCG day	4
High dose, follicular phase protocol (F)	More than 8	From follicular phase of the stimulation cycle to hCG day	2
High dose, long-term protocol (G)		For at least 2 weeks, always from preceding cycle to hCG day	5

### Methodological quality assessment

We utilized stringent quality assessment methodologies to substantiate the integrity of the outcomes. Among the included RCTs, 9 (60.0%) trials had a low risk of bias on random sequence generation and 7 (46.7%) trials were with a low risk of bias on allocation concealment. No study had a high risk for bias on random sequence generation and allocation concealment. Only 6 (40.0%) trials had a low risk of bias on both blinding of participants and personnel and outcome assessment. There were 14 (93.3%) RCTs with low risk and one study with high risk of incomplete outcome data. The risk of bias due to selective reporting in the literature was unclear. One study was at high risk of bias due to insufficient patient recruitment, while other studies were at low or unclear risk. As for cohort studies, the NOS scores ranged from 8 to 9. The methodological quality assessments of studies are summarized in Supplementary Figure S1.

### Publication bias assessment

A funnel plot was constructed to assess publication bias for the primary outcome measure. The results showed that the majority of points fall within the funnel, with a symmetrical distribution on both sides, suggesting a lower likelihood of publication bias (Supplementary Figure S2).

### Inconsistency testing results

**Figure 2:**
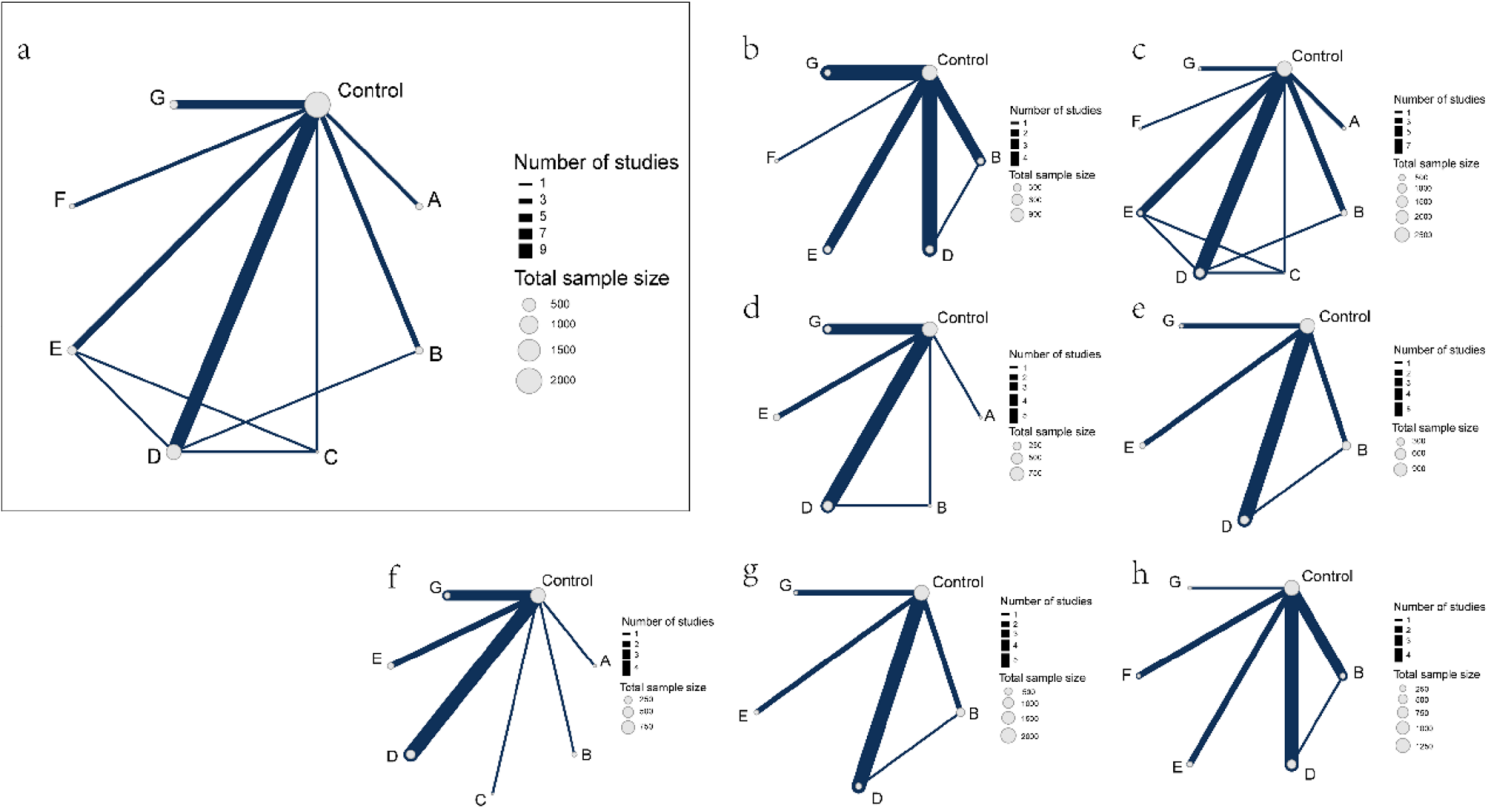
Network map of different GH supplementation protocol comparisons. The size of the nodes describes the total sample size of GH supplementation protocols. The thickness of the lines shows the number of studies. (**a**) Clinical pregnancy rate. (**b**) Total dosage of Gn required for ovarian stimulation. (**c**) Number of retrieved oocytes. (**d**) Number of retrieved MII oocytes. (**e**) Number of fertilized embryos. (**f**) Serum E2 levels on hCG day. (**g**) Endometrial thickness on hCG day. (**h**) Live birth rate.

The network maps for the primary and secondary outcome measures can be found in Fig. 2. Global inconsistency was tested for all outcomes. The results showed that there was no inconsistency in any outcome measures, so a consistency model was selected for data synthesis (Supplementary Table S1). Local inconsistency was further evaluated for outcomes with closed loops in the network and the results showed no local inconsistency in outcome measures except for clinical pregnancy rate (Supplementary Table S2).

### NMA results

#### Primary outcome measure: clinical pregnancy rate

**Figure 3:**
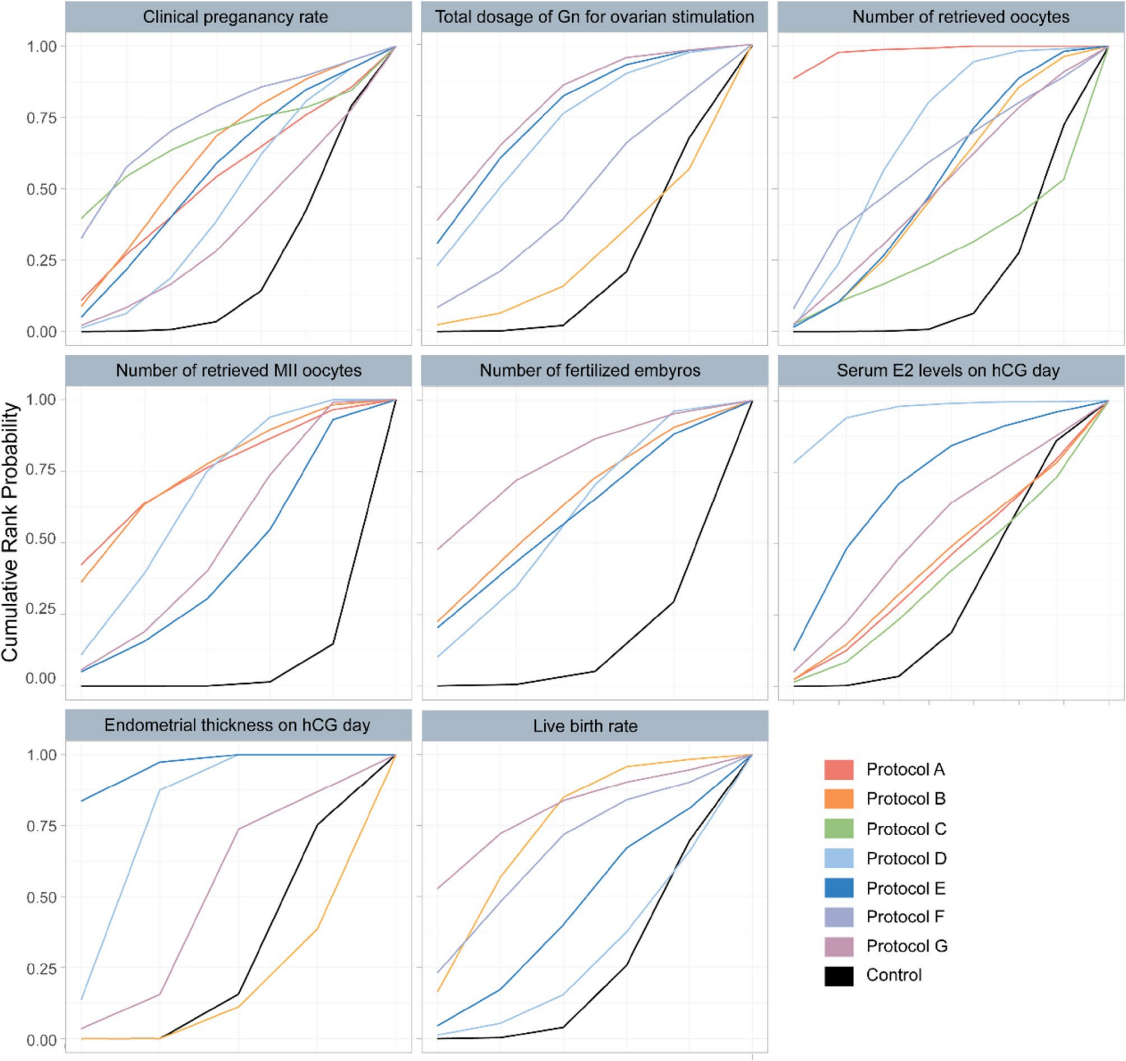
The ranking of different GH supplementation protocols according to the surface under the cumulative ranking curve (SUCRA) value for primary and secondary outcomes.

All studies^29–51^ reported the clinical pregnancy rate for seven different GH supplementation protocols. Results showed that no protocol could significantly improve the clinical pregnancy rate in POR patients (compared to the control group, > 0.05) (Table 3). The SUCRA values for different GH protocols were as follows: F (0.72), C (0.66), B (0.56), E (0.56), A (0.52), D (0.43), G (0.35), Control (0.21) (Fig. 3).

**Table 3:** ORs and MDs with 95%Crls of NMA for the primary and secondary outcome measures.

Clinical pregnancy rate							
A							
0.37 (0.06, 2.17)	B						
1.44 (0.32, 6.64)	3.97 (0.66, 24.61)	C					
0.79 (0.24, 2.75)	2.17 (0.55, 9.41)	0.55 (0.16, 1.90)	D				
1.02 (0.33, 5.08)	2.84 (0.63, 19.32)	0.72 (0.02, 3.46)	1.29 (0.55, 4.60)	E			
0.26 (0.01, 3.47)	0.72 (0.02, 11.94)	0.18 (0.00, 2.52)	0.34 (0.01, 3.68)	0.25 (0.01, 2.65)	F		
1.16 (0.37, 4.66)	3.23 (0.70, 18.71)	0.82 (0.23, 3.40)	1.48 (0.61, 4.23)	1.14 (0.33, 3.06)	4.45 (0.41, 162.05)	G	
1.33 (0.53, 4.25)	3.67 (0.94, 18.09)	0.94 (0.31, 3.11)	1.69 (0.96, 3.48)	1.31 (0.49, 2.67)	5.02 (0.53, 175.42)	1.44 (0.55, 2.35)	Control

Total dosage of Gn for ovarian stimulation							
B							
245.89 (− 786.49, 1273.94)	D						
− 111.69 (− 1363.83, 1127.48)	− 356.84 (− 1535.82, 821.87)	E					
− 113.47 (− 1909.22, 1679.22)	− 358.14 (− 2122.77, 1402.79)	− 1.78 (− 1833.88, 1834.70)	F				
− 212.72 (− 1366.69, 935.47)	− 458.44 (− 1537.89, 622.59)	− 102.10 (− 1288.64, 1097.42)	− 101.64 (− 1869.19, 1667.54)	G			
− 519.58 (− 1373.20, 331.73)	**− 764.31 (− 1519.71, − 12.46)**	− 408.94 (− 1309.38, 503.63)	− 407.25 (− 1992.87, 1183.49)	− 305.46 (− 1082.92, 469.57)	Control		

Number of retrieved oocytes							
A							
2.44 (0.25, 4.57)	B						
3.22 (0.06, 6.52)	0.79 (− 2.11, 3.84)	C					
2.00 (0.07, 3.96)	− 0.44 (− 1.82, 1.05)	− 1.23 (− 3.98, 1.45)	D				
2.39 (0.30, 4.52)	− 0.05 (− 1.73, 1.73)	− 0.84 (− 3.65, 1.91)	0.39 (− 1.01, 1.79)	E			
2.09 (− 0.96, 5.10)	− 0.35 (− 3.11, 2.43)	− 1.14 (− 4.88, 2.47)	0.085 (− 2.56, 2.66)	− 0.30 (− 3.09, 2.40)	F		
2.42 (− 0.03, 4.81)	− 0.02 (− 2.11, 2.08)	− 0.81 (− 4.08, 2.32)	0.42 (− 1.52, 2.25)	0.03 (− 2.07, 2.03)	0.33 (− 2.64, 3.31)	G	
**3.18 (1.42, 4.92)**	0.75 (− 0.51, 2.02)	− 0.05 (− 2.84, 2.62)	**1.19 (0.29, 2.00)**	0.80 (− 0.43, 1.96)	1.10 (− 1.36, 3.57)	0.77 (− 0.90, 2.44)	Control

Number of retrieved MII oocytes							
A							
0.09 (− 3.62, 3.79)	B						
0.45 (− 2.60, 3.51)	0.36 (− 2.02, 2.78)	D					
1.09 (− 2.23, 4.48)	1.01 (− 1.99, 4.07)	0.64 (− 1.51, 2.82)	E				
0.53 (− 2.68, 3.78)	0.44 (− 2.40, 3.32)	0.07 (− 1.85, 2.02)	− 0.56 (− 2.99, 1.82)	G			
2.28 (− 0.52, 5.13)	2.19 (− 0.19, 4.62)	**1.82 (0.68, 2.99)**	1.19 (− 0.66, 3.01)	**1.75 (0.19, 3.31)**	Control		

Number of fertilized embryos							
B							
0.21 (− 2.19, 2.70)	D						
0.10 (− 3.10, 3.37)	− 0.11 (− 2.84, 2.66)	E					
− 0.59 (− 3.80, 2.70)	− 0.81 (− 3.58, 2.00)	− 0.70 (− 4.02, 2.63)	G				
1.29 (− 0.91, 3.57)	1.07 (− 0.38, 2.55)	1.18 (− 1.16, 3.52)	1.88 (− 0.48, 4.26)	Control			

Serum E2 levels on hCG day							
A							
264.36 (− 1704.53, 2227.89)	B						
742.49 (− 1242.30, 2716.70)	478.17 (− 1496.06, 2433.95)	C					
− 81.83 (− 1642.86, 1476.21)	− 348.13 (− 1885.27, 1205.20)	− 821.18 (− 2380.80, 753.53)	D				
− 71.07 (− 1788.63, 1626.38)	− 338.37 (− 2025.04, 1346.29)	− 813.18 (− 2515.58, 894.48)	8.51(− 1190.58, 1196.47)	E			
82.23 (− 1552.21, 1677.21)	− 180.66 (− 1816.73, 1399.80)	− 658.57 (− 2303.33, 944.33)	162.54 (− 926.39, 1208.77)	153.53 (− 1131.86, 1399.72)	G		
487.04 (− 915.80, 1879.14)	221.07 (− 1158.47, 1599.61)	− 254.15 (− 1657.64, 1148.68)	567.31 (− 128.15, 1252.70)	557.25 (− 414.27, 1533.30)	403.97 (− 390.68, 1238.57)	Control	

Endometrial thickness on hCG day							
B							
− 0.50 (− 0.88, − 0.12)	D						
− 0.71 (− 1.28, − 0.23)	− 0.21 (− 0.74, 0.23)	E					
− 0.24 (− 0.77, 0.35)	0.26 (− 0.24, 0.81)	0.47 (− 0.09, 1.16)	G				
− 0.06 (− 0.40, 0.26)	**0.44 (0.18, 0.68)**	**0.64 (0.27, 1.10)**	0.18 (− 0.31, 0.61)	Control			

Live birth rate							
B							
1.70 (0.62, 7.22)	D						
1.36 (0.40, 7.26)	0.80 (0.19, 3.36)	E					
0.98 (0.17, 4.30)	0.57 (0.08, 2.20)	0.72 (0.09, 3.08)	F				
0.65 (0.10, 5.38)	0.38 (0.05, 2.50)	0.47 (0.06, 3.47)	0.66 (0.09, 8.19)	G			
1.73 (0.88, 5.85)	1.02 (0.42, 2.67)	1.27 (0.44, 4.01)	1.79 (0.59, 10.75)	2.70 (0.53, 16.73)	Control		

Significant values are in bold.

#### Secondary outcome measure: total dosage of Gn required for ovarian stimulation

Fourteen studies^33–36,38,40–42,44–46,48,50,51^ reported the total Gn dosage for five different GH supplementation protocols. Protocol D was found to significantly reduce the Gn dosage in POR patients (compared to the control group, MD − 764.31, 95%CrI − 1519.70 to − 12.46) (Table 3). The SUCRA values for different GH protocols were as follows: D (0.78), B (0.60), E (0.52), F (0.51), G (0.45), Control (0.16) (Fig. 3).

#### Secondary outcome measure: number of retrieved oocytes

Seventeen studies^30,34–36,38–49,51^ reported the number of retrieved oocytes for seven different GH supplementation protocols. Protocol A and Protocol D were found to significantly increase the number of retrieved oocytes in POR patients (compared to the control group, MD 3.18, 95%CrI 1.42 to 4.92; MD 1.19, 95%CrI 0.29 to 2.00, respectively) (Table 3). The SUCRA values for different GH protocols were as follows: A (0.98), D (0.64), F (0.56), E (0.48), G (0.47), B (0.46), C (0.26), Control (0.15) (Fig. 3).

#### Secondary outcome measure: number of retrieved MII oocytes

Twelve studies^33–36,38–40,42,46,48–50^ reported the number of retrieved MII oocyte for five different GH supplementation protocols. Protocol D and Protocol G were found to significantly increase the number of retrieved MII oocytes in POR patients (compared to the control group, MD 1.82, 95%CrI 0.68 to 2.99; MD 1.75, 95%CrI 0.19 to 3.31, respectively) (Table 3). The SUCRA values for different GH protocols were as follows: B (0.72), A (0.72), D (0.64), G (0.48), E (0.40), Control (0.03) (Fig. 3).

#### Secondary outcome measure: number of fertilized embryos

Ten studies^33–36,38,42,44,46,48,49^ reported the fertilized embryo count for four different GH supplementation protocols. Results showed that no protocol could significantly improve the number of fertilized embryos in POR patients (compared to the control group, *p* > 0.05) (Table 3). The SUCRA values for different GH protocols were as follows: G (0.76), B (0.59), E (0.54), D (0.52), Control (0.09) (Fig. 3).

#### Secondary outcome measure: serum E2 levels on hCG day

Twelve studies^33–40,42,43,48,49^ reported the serum E2 levels on hCG day for six different GH supplementation protocols. Results showed that no protocol could significantly improve the serum E2 levels on hCG day in POR patients (compared to the control group, *p* > 0.05) (Table 3). The SUCRA values for different GH protocols were as follows: D (0.70), E (0.68), A (0.61), G (0.58), B (0.46), Control (0.26), C (0.22) (Fig. 3).

#### Secondary outcome measure: endometrial thickness on hCG day

Ten studies^34–36,38,40,42,46–49^ reported the endometrial thickness on hCG day for four different GH supplementation protocols. Protocol D and Protocol E were found to significantly increase the endometrial thickness in POR patients (compared to the control group, MD 0.44, 95%CrI 0.18 to 0.68; MD 0.64, 95%CrI 0.27 to 1.10, respectively) (Table 3). The SUCRA values for different GH protocols were as follows: E (0.94), D (0.75), G (0.45), Control (0.23), B (0.12) (Fig. 3).

#### Secondary outcome measure: live birth rate

Eleven studies^29,31,38,41,42,44–47,50,51^ reported the live birth rate for five different GH supplementation protocols. None of the protocols showed a significant improvement in the live birth rate in POR patients (compared to the control group, *p* > 0.05) (Table 3). The SUCRA values for different GH protocols were as follows: G (0.80), B (0.67), F (0.64), E (0.43), D (0.25), Control (0.20) (Fig. 3).

### Comprehensive ranking results

The comprehensive ranking results for all the outcomes are displayed in Table 3. The asterisk (*) indicated results that had a statistically significant difference compared to the control group (*p* < 0.05), while the remaining treatment protocols showed no statistically significant difference (*p* > 0.05) (Table 4).

**Table 4:** A comprehensive sorting table for the primary and secondary outcome measures.

No.	Clinical pregnancy rate	Total dosage of Gn	Number of retrieved oocytes	Number of retrieved MII oocytes	Number of fertilized embryos	Serum E2 levels on hCG day	Endometrial thickness on hCG day	Live birth rate
1	F	D*	A*	B	G	D	E*	G
2	C	B	D*	A	B	E	D*	B
3	B	E	G	D*	E	G	G	F
4	E	F	E	G*	D	A	Control	E
5	A	G	B	E	Control	B	B	D
6	D	Control	C	Control	–	Control	–	Control
7	G	–	Control	–	–	C	–	–
8	Control	–	–	–	–	–	–	–

### Subgroup analysis

According to the NMA's results, medium dose, follicular phase protocol (Protocol D) was utilized the most frequently. In addition, it demonstrated greater efficacy than other protocols in terms of minimizing the total dose of Gn, increasing the number of retrieved oocytes, and enhancing endometrial conditions with a moderate administration time and injection dose. Therefore, we conducted a subgroup analysis based on diagnostic criteria, study design, COH protocol, and geographic region, focusing on the primary outcome measure (clinical pregnancy rate), to explore potential sources of heterogeneity among studies (the study by Dor^31^ was not included in the analysis as both the experimental and control groups had zero events, not contribute to the meta-analysis). The results showed that Protocol D significantly improved the clinical pregnancy rate in the RCT subgroup (OR 1.76, 95% CI 1.12 to 2.76, *p* = 0.014, I^2^ = 0.0). In addition, trials in Africa showed relatively positive therapeutic effects (OR 1.65, 95% CI 1.03 to 2.65, *p* = 0.039, I^2^ = 0.0). It did not show a significant improvement in the clinical pregnancy rate in the remaining subgroups (*p* > 0.05) (Table 5).

**Table 5:** Subgroup analysis of the impact of medium dose, follicular phase protocol on clinical pregnancy rate in patients with POR.

Outcomes	Number of studies	Sample size	OR (95%CI)	I^2^(%)	*P* value
Diagnostic criteria (random effect model)					
The Bologna criteria	4	622	1.16 (0.78, 1.74)	58.9	0.459
The Poseidon Criteria (P4)	1	1120	0.92 (0.68, 1.24)	0.0	0.591
Self-defining criteria	3	166	1.51 (0.69, 3.32)	0.0	0.304
Study design (fix effect model)					
RCT	6	487	1.76 (1.12, 2.76)	0.0	**0.014**
Non-RCT	2	1421	0.85 (0.65, 1.12)	20.90	0.252
COH protocol (fix effect model)					
GnRHant	2	176	1.86 (0.83, 4.15)	0.0	0.132
GnRHant ultrashort	1	140	1.41 (0.60, 3.34)	0.0	0.432
GnRHa short	3	171	1.96 (0.97, 3.95)	0.0	0.061
GnRHa long	1	301	0.61 (0.32, 1.17)	0.0	0.137
Multiple protocols	1	1120	0.92 (0.68, 1.24)	0.0	0.591
Region (fix effect model)					
Europe	2	261	2.13 (0.29, 15.54)	0.0	0.456
Asia	3	1481	0.88 (0.67, 1.15)	39.1	0.358
Africa	3	401	1.65 (1.03, 2.65)	0.0	**0.039**

Significant values are in bold.

## Discussion

With an increasing number of women delaying childbirth, POR has become a great obstacle in ART, which could lead couples to abandon treatment or seek oocyte donation^20^. GH has a long-standing history of use as an adjunctive therapy in the treatment of POR. However, there is no standardized protocol for the application of GH to date and the clinical efficacy of GH remains controversial^52^. Thus, we endeavor to explore whether there is an optimal GH supplementation protocol for enhancing the clinical outcomes of POR patients. This study represents a comprehensive synthesis of data regarding currently GH supplementation protocols for PORs undergoing ART therapy. The results of this NMA can be summarized as follows. First, administering 4–8 IU/d of GH during the follicular phase of the stimulation cycle was the most commonly used protocol in the clinic and it resulted in the best comprehensive improvement in clinical outcomes for POR patients, including increasing the number of retrieved oocytes, the number of mature oocytes, endometrial thickness on hCG day, and reducing total Gn requirements, but had no effect on the number of fertilized embryos, serum E2 levels on hCG day, live birth rate per embryo transfer cycle. Second, subgroup analysis focus on the clinical pregnancy rate showed that the aforementioned treatment protocol could significantly improve the clinical pregnancy rate of POR patients in the RCT subgroup and the African subgroup, but diagnostic criteria and ovarian stimulation protocols appear to not correlate with the efficacy of this protocol. Third, using GH for at least 2 weeks before oocyte retrieval has certain benefits in improving the number of oocytes retrieved and endometrial thickness on the day of hCG administration, however, the advantages are not significantly greater than those of the former protocol, and the optimal dosage remains unclear; therefore, further research is warranted.

Only a handful of prior meta-analyses examined the differences in efficacy among different GH administration protocols on assisted reproductive outcomes in POR patients. Based on the timing of GH administration, Yu et al.^21^ categorized GH protocols into luteal phase protocols and follicular phase protocols, and the results demonstrated that only GH administration during the follicular phase improved pregnancy rates and live birth rates in patients with POR. Similarly, Cozzolino et al.^53^ analyzed the influence of GH administration timing on clinical pregnancy rate of PORs and found that positive result was observed only when it was used during the ovarian stimulation period. Nevertheless, neither of the above studies accounted for GH dosage. Shang and colleagues^19^ also confirmed a dose- and time-dependent association between GH and clinical outcomes in POR patients. They indicated that daily administration of < 5 IU/d of GH or administration starting from the follicular phase of the previous cycle before COH increased endometrial thickness and the likelihood of conception more significantly. In contrast, administration of 5–10 IU/day was associated with better oocyte and embryo quality. However, in this report, they only used traditional meta-analysis methods and treated daily dosage and administration timing of GH as two independent variables, which were not conducive to determining the optimal GH administration protocol.

In response to the aforementioned issues, we placed a significant emphasis on additional research. To our knowledge, this is the first meta-analysis to classify GH supplementation protocols by integrating daily dosage and schedule of administration. In accordance with previous research^22,53^, our findings demonstrate the efficacy of adding GH during the follicular phase of the stimulation cycle. The present NMA indicates that a dosage of 4–8 IU/day of GH administered during the follicular phase of stimulation cycle is the most widely used protocol in clinical practice and has the best comprehensive therapeutic effects on improving the number of oocytes and endometrial thickness, which were regarded two crucial factors for increasing embryo implantation rates and pregnancy rates in assisted reproduction, and reducing the total dose of Gn for ovarian stimulation, which was considered to be one of the important indicators to evaluate ovarian response. In addition to its clinical efficacy, this method also offers the benefits of a shorter administration time, a more moderate injection dosage, and a more convenient injection process, which can alleviate the inconvenience and pain associated with frequent clinic injections, as well as the financial burden on patients. Thus, this protocol seems to be the optimal protocol and has broad applications and research prospects. However, we did not observe the impact of this protocol on fertilized embryo number and E2 level on the hCG day, which may be related to the inconsistent POR diagnostic criteria of the involved patients and the inclusion of non-RCTs, therefore, the results of those endpoints need further confirmation.

Previous meta-analyses have consistently shown controversial results regarding the effect of adding GH on the clinical pregnancy rate and live birth rate of patients with POR. A traditional meta-analysis by Kolibianakis et al.^20^ suggested that the addition of GH could improve the probability of clinical pregnancy and live birth in POR patients. Another meta-analysis by Yu et al.^21^ indicated that the clinical pregnancy rate of POR patients was not associated with the addition of GH. Meta-analysis by Shang et al.^19^ reported that adding GH could improve clinical pregnancy rates and live births in POR patients. The recent meta-analysis by Cozzolino et al.^53^ showed that supplementation of GH in POR patients increased the clinical pregnancy rate, but did not affect the ongoing pregnancy rate and live birth rate. Some single studies have also drawn inconsistent conclusions^38,49,54^. In our NMA, no GH supplementation protocol has been proven effective in improving the clinical pregnancy rate and live birth rate of POR patients according to the overall pooled results. However, it is important to consider that the objective of COH is to recruit a sufficient number of follicles to compensate for the inefficiencies of embryo culture and selection for transfer^55^, and in assisted reproduction, the number of retrieved oocytes is regarded as a significant prognostic variable with a linear correlation to live birth rates^56^, so it is possible that, despite the lack of improvement in pregnancy rate and live birth rate within a single transfer cycle, the increased quantity and maturity of oocytes may contribute to a greater likelihood of cumulative pregnancies and live births due to more embryos available for transfer and cryopreservation. We look forward to conducting further research in the future to validate this hypothesis.

Medium dose, follicular phase protocol (Protocol D) is extensively employed and relatively effective, so it was the focus of subgroup analyses. We hypothesized, based on existing research, that diverse diagnostic criteria, ovarian stimulation protocols, geographical regions, and ethnicities may contribute to the heterogeneity. In addition, since non-RCTs were included, subgroups were also categorized according to the study design. The results showed low heterogeneity (I^2^ < 50%) in most subgroups, indicating that the aforementioned factors are likely the main contributors to heterogeneity. We found that when only analyzing RCTs, Protocol D could significantly increase the clinical pregnancy rate of patients, but the overall pooled results showed the opposite effect, partially confirming the efficacy of this protocol but also indicating the uncertainty of the results, which highlights the importance of conducting high-quality RCTs. This protocol received more positive feedback in studies conducted in Africa, which may be attributed to the fact that all African studies were RCTs published within the past decade. Diagnostic criteria appeared to not correlate with the efficacy of Protocol D on clinical pregnancy rate, but standardized diagnostic criteria is still critical because it can effectively reduce population heterogeneity and improve the repeatability of results. Moreover, efficacy of Protocol D on primary outcome measures seems unaffected by the COH regimen. Although the recent studies we included tended to adopt gonadotropin-releasing hormone (GnRH) antagonist protocol, this may not be generalizable. Current COH protocols for PORs mainly include GnRH antagonist protocol, progestion-primed ovarian stimulation (PPOS) protocol, oral superovulation agent protocol, GnRH agonist long protocol, natural/modified-natural cycle protocol, etc.^57–60^. Recent studies seem to focus more on the former two protocols. However, the absence of clear evidence indicating which protocol is preferable means that clinical practice relies to some extent on the experience^9^. A latest meta-analysis of GH supplementation in women with diminished ovarian reserve reported that GH, when used in conjunction with the GnRH antagonist protocol, resulted in a greater increase in the number of oocytes retrieved compared with the GnRH agonist protocol^61^. But there is still no direct evidence to prove the superiority or inferiority of different COH protocols when GH is used as an adjuvant therapy in POR treatment. The above issues require further exploration. Owing to the small sample sizes, results of subgroup analysis should be interpreted with caution.

It is commonly accepted that the development and maturation of follicles require approximately 75 days, and the role of GH is found to begin at the initial stage of follicle development^62,63^. Therefore, to enhance oocyte yield, it seems more appropriate to initiate GH co-treatment before commencing ovarian stimulation, which is consistent with the regularity of follicle growth and maturation. Gleicher and colleagues endorsed this hypothesis and suggested that the administration of GH should at least 6 weeks prior to COH^64^. In this NMA, we have found that compared to the control group, using GH at a dosage > 8 IU/day for at least 2 weeks before oocyte retrieval could improve the number of obtained oocytes in POR patients but did not show significant advantages compared to the medium dose, follicular phase protocol (protocol D). Therefore, the application value of this protocol needs further exploration. We have also found that the administration of GH at a dosage of 4-8 IU/day for at least 2 weeks had advantages in improving endometrial thickness on hCG day (an important indicator for assessing endometrial receptivity^65^), which may provide an alternative treatment option for POR patients who have thin endometrium or poor endometrial receptivity, with the goal of improving the implantation rate. Besides, some existing studies have focused on the efficacy of administrating GH at a dosage less than 4 IU/day (especially < 1 IU/day), which is considered low-dose, for at least 2 weeks. Lattes et al.^66^ and Safdarian et al.^46^ both reported that administration of GH of 0.3–0.5 IU/day from the preceding cycle to hCG day had positive effects on ovarian stimulation and pregnancy outcomes. If this protocol is proven effective, its advantages in reducing GH dosage and lowering medical expenses may position it as a promising treatment regimen in the future. Unfortunately, the current evidence from our NMA do not support the widespread application of the low-dose, long-term protocol in clinical practice, and more research is required to further evaluate its effectiveness and advantages.

Other protocols are utilized on a relatively infrequent basis, and they have not demonstrated any advantages in terms of efficacy or cost-effectiveness, indicating limited clinical viability. However, sample size and study design affect the quality of evidence, so the results need to be interpreted rationally.

It should be noted that none of the RCTs included in this NMA had a high risk of bias for random sequence generation and allocation concealment, and only one had a high risk of incomplete outcome data (the three items mentioned above are deemed to be the key entries^67^). Additionally, the cohort studies included in this NMA had NOS scores of 8–9. In general, included clinical had a moderately good design and methodological quality. Thus, the findings of our NMA can provide some evidence for clinicians when making decisions about the timing and dosage of GH administration in patients with POR. Nonetheless, as a result of the limited number, small scale and unavoidable clinical heterogeneity of extant clinical studies, the level of existing evidence is not very strong, and more well-designed trials would be necessary to determine the application value of these protocols.

This study has some limitations that should be considered. First, despite undertaking subgroup analyses, there was still substantial heterogeneity within some subgroups, which may be associated with differences in outcomes across reproductive medicine centers. Due to the small number of available studies, we did not further conduct a meta-regression analysis. Second, there were a few indicators with local inconsistency in our NMA, which may reduce the level of evidence. Third, similar to most of the previous studies, the cost-effectiveness ratio of various protocols was not evaluated, thus, it was not possible to provide clinical recommendations that were appropriate from a health economics standpoint. Fourth, an evaluation of safety, including adverse reactions during medication, risk of birth defects, long-term health status of both mothers and progenies with the use of GH co-treatment were not conducted due to insufficient pertinent data. Fifth, the included studies exhibited clinical heterogeneity due to the diverse COH protocols used, which may have influenced the results. However, limited by the number of eligible studies, we failed to perform a NMA by COH protocol to eliminate this confounder and were unable to further explore whether adding GH has different efficacy in different COH protocols. Sixth, the study by Choe et al.^40^ used a growth hormone sustained release agent and we categorized it as a high-dose, long-term protocol. However, the reasonableness of this classification method remains to be further explored.

In the future, it would be necessary to conduct larger-scale multi-arm parallel RCTs with rigorous design using the Bologna criteria or the Poseidon criteria to validate the clinical efficacy, cost-effectiveness, and safety profile of various GH supplementation protocols and to provide additional evidence for clinical decision-making. Besides, it would be meaningful to investigate whether the type of COH protocol affects the efficacy of GH supplementation therapy. In addition, further research is warranted to explore the factors which can predict the efficacy of GH, so that to standardize the indications for incorporating GH in clinical practice.

## Conclusions

The use of a GH supplementation protocol with a daily dosage of 4-8 IU during the follicular phase of the stimulation cycle has the best comprehensive therapeutic effects on improving clinical outcomes in women with POR undergoing ART therapy with a relatively brief treatment duration and a moderate total GH dose. Therefore, its clinical application is suggested. Besides, the potential advantages of long-term GH supplementation protocol (using GH for at least 2 weeks before oocyte retrieval) has merit for further research. Future clinical trials of higher quality are required to compare the clinical efficacy, cost-effectiveness ratio and safety of various GH supplementation protocols to provide patients with more precise treatment guidance.

### Supplementary Information

Supplementary Figures.

Supplementary Tables.

## Data Availability

The datasets generated during and/or analyzed during the current study are available throughout the manuscript.

## Abbreviations

ART: Assisted reproductive technology
Co Q10: Coenzyme Q10
COH: Controlled ovarian hyperstimulation
DHEA: Dehydroepiandrosterone
DIC: Deviation information criterion
GH: Growth hormone
Gn: Gonadotropin
GnRH: Gonadotropin-releasing hormone
hCG: Human chorionic gonadotropin
ICSI: Intracytoplasmic sperm injection
IGF: Insulin-like growth factor
IVF: In vitro fertilization
MD: Mean difference
NMA: Network meta-analysis
NOS: Newcastle-Ottawa Scale
OR: Odds ratio
POR: Poor ovarian response
PRISMA-NMA: Preferred Reporting Items for Systematic Reviews for Network Meta-Analysis
RCT: Randomized controlled trials
rLH: Recombinant luteinizing hormone
SUCRA: Surface under the cumulative ranking curve
