# Supplementary material for: Comparative efficacy of different growth hormone supplementation protocols in improving clinical outcomes in women with poor ovarian response undergoing assisted reproductive therapy: a network meta-analysis
Source: Sci Rep. 2024 Feb 9;14:3377. doi: 10.1038/s41598-024-53780-z (PMC10858197; doi:10.1038/s41598-024-53780-z)

**Supplementary Figure S1.** Quality evaluation of included trials. a, The Cochrane Risk of Bias Tool for assessing the quality of RCTs. b, The Newcastle-Ottawa Scale for assessing the quality of cohort studies.


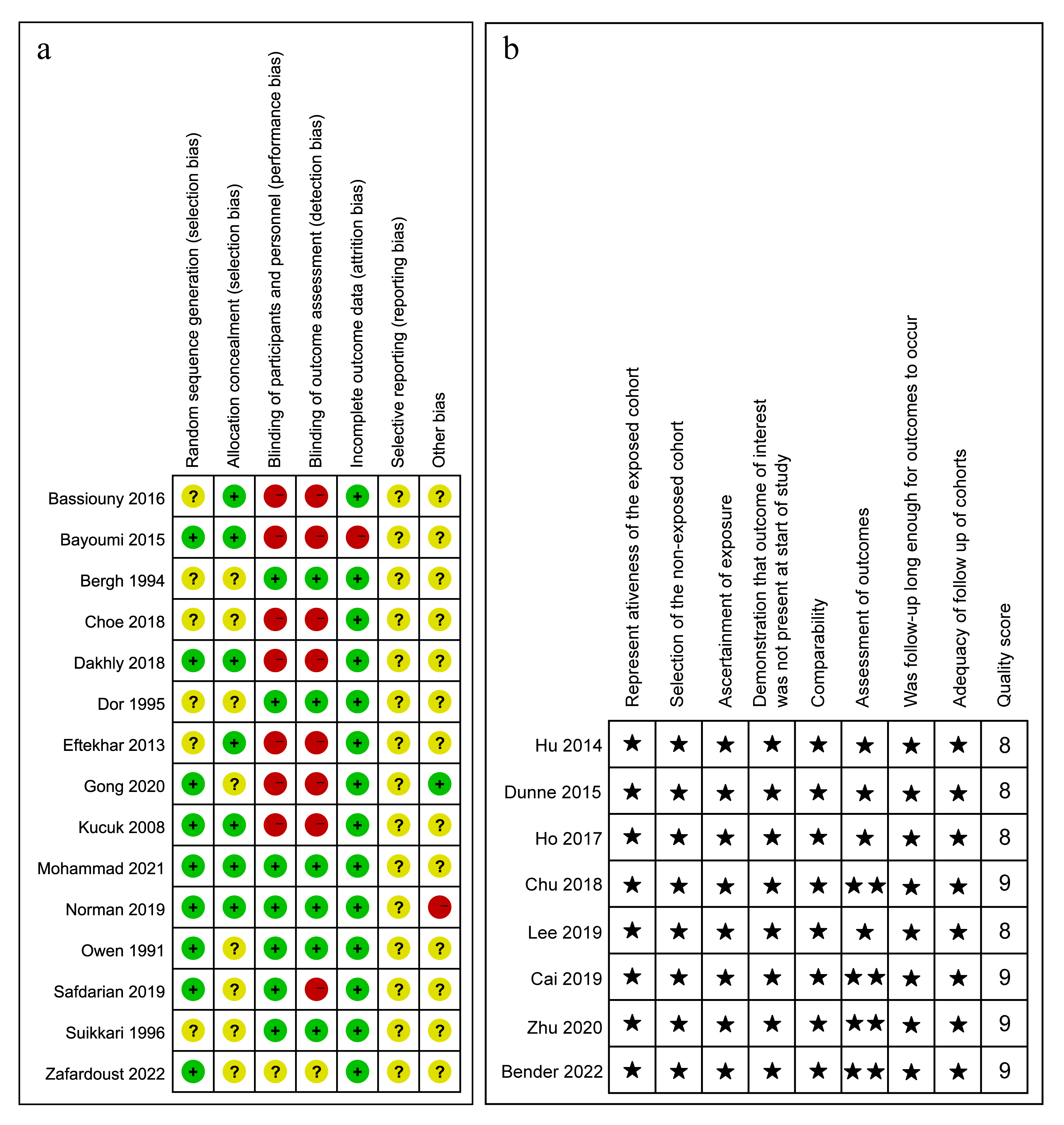


**Supplementary Figure S2.** Funnel plot for the clinical pregnancy rate.


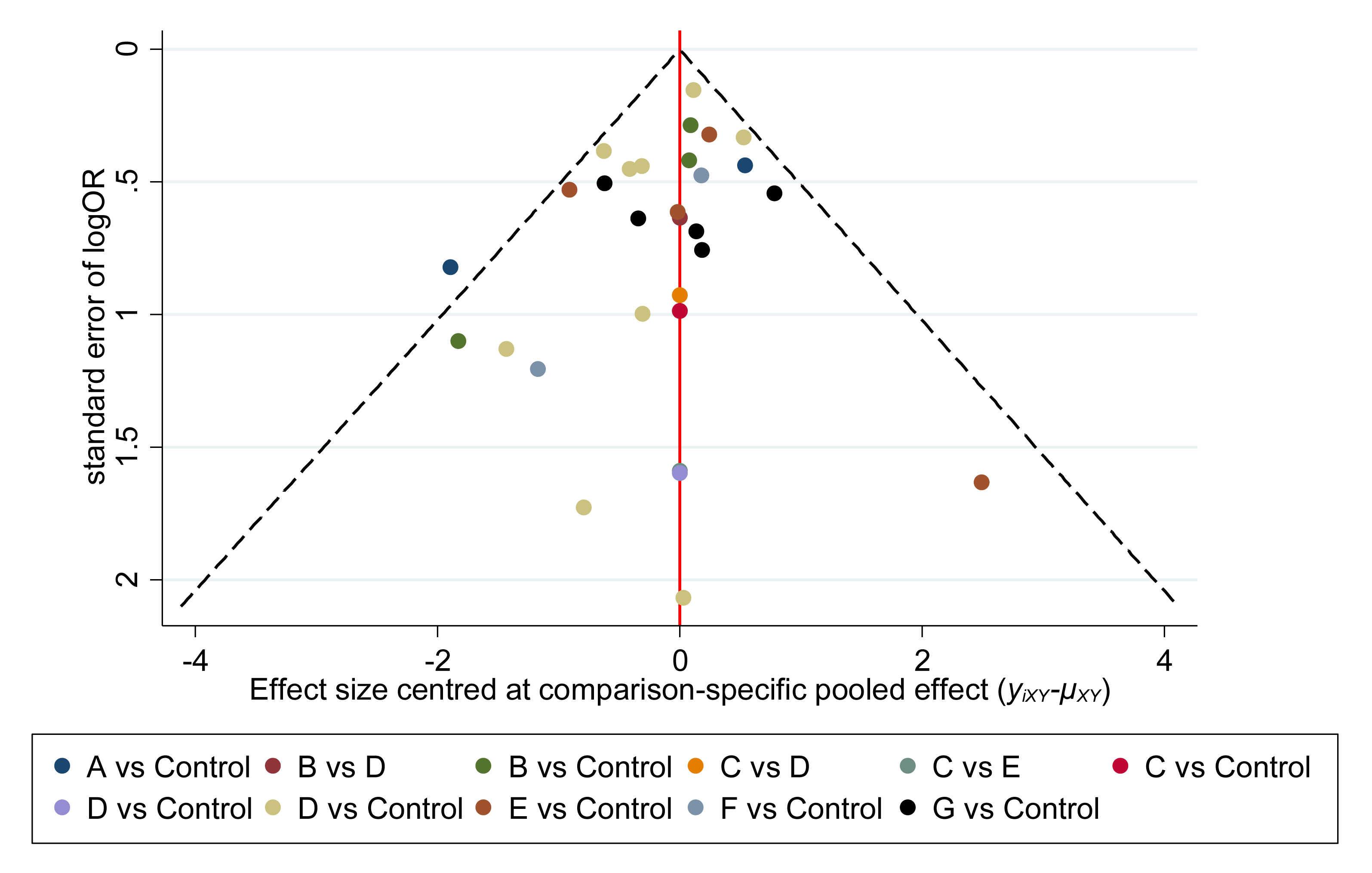

Supplement: Supplementary file 1 — Supplementary Figures. [file 41598_2024_53780_MOESM1_ESM.docx]
