# Supplementary material for: Comparative efficacy of different growth hormone supplementation protocols in improving clinical outcomes in women with poor ovarian response undergoing assisted reproductive therapy: a network meta-analysis
Source: Sci Rep. 2024 Feb 9;14:3377. doi: 10.1038/s41598-024-53780-z (PMC10858197; doi:10.1038/s41598-024-53780-z)
Supplement: Supplementary file 2 — Supplementary Tables. [file 41598_2024_53780_MOESM2_ESM.docx]

**Supplementary Table S1.** Global inconsistency testing results: comparison of DIC values of consistency model and inconsistency model.

| **Outcome Measures** | **DIC values** | | **Difference of DIC values between two models** | **Model selection** |
| --- | --- | --- | --- | --- |
|  | Consistent model | Inconsistent model |  |  |
| Clinical pregnancy rate | 90.7 | 90.6 | 0.1 | Consistent model |
| Total dosage of Gn required for ovarian stimulation | 3546.9 | 3548.9 | 2.0 | Consistent model |
| Number of retrieved oocytes | 72.8 | 72.7 | 0.1 | Consistent model |
| Number of retrieved M2 oocytes | 49.3 | 48.8 | 0.5 | Consistent model |
| Number of fertilized embryos | 41.9 | 41.3 | 0.6 | Consistent model |
| Serum E2 levels on hCG day | 306.2 | 305.4 | 0.8 | Consistent model |
| Endometrial thickness on hCG day | 37.2 | 36.8 | 0.4 | Consistent model |
| Live birth rate | 41.0 | 40.8 | 0.2 | Consistent model |

**Supplementary Table S2.** Local inconsistency testing results: direct and indirect comparison from the network meta-analysis (NMA) and the inconsistency test results.

| **Comparison** | **NMA** | **Direct** | **Indirect** | **Difference** | **Diff. lower 95%CI** | **Diff. upper 95%CI** | ***P*-value** |
| --- | --- | --- | --- | --- | --- | --- | --- |
| **Clinical pregnancy rate** | | | | | | | |
| D vs B | -0.24 | -0.18 | -0.27 | 0.09 | -1.89 | 2.05 | 0.94 |
| E vs D | 0.12 | -9.09 | 0.49 | -9.58 | -23.27 | -1.46 | **<0.01** |
| **Total dosage of Gn required for ovarian stimulation** | | | | | | | |
| D vs B | 17.13 | 13.86 | 17.15 | -3.29 | -22.48 | 15.95 | 0.73 |
| **Number of retrieved oocytes** | | | | | | | |
| D vs B | 0.41 | 0.56 | 0.33 | 0.23 | -2.05 | 2.69 | 0.85 |
| E vs D | -0.37 | -0.89 | -0.25 | -0.64 | -3.93 | 2.53 | 0.70 |
| **Number of fertilized embryos** | | | | | | | |
| D vs B | -0.21 | 0.44 | -0.68 | 1.12 | -2.43 | 4.74 | 0.49 |
| **Endometrial thickness on hCG day** | | | | | | | |
| D vs B | 0.50 | 0.63 | 0.40 | 0.23 | -0.40 | 0.86 | 0.41 |
| **Live birth rate** | | | | | | | |
| D vs B | -0.78 | -1.27 | -0.71 | -0.57 | -4.48 | 3.22 | 0.71 |
